# Long-Life Inoculant: *Bradyrhizobium* Stored in Biodegradable Beads for Four Years Shows Optimal Cell Vitality, Interacts with Peanut Roots, and Promotes Early Growth

**DOI:** 10.3390/plants13212983

**Published:** 2024-10-25

**Authors:** Adriana Belén Cesari, Marilina Fernandez, Natalia Soledad Paulucci, Marta Susana Dardanelli

**Affiliations:** 1 Departamento de Biología Molecular, Facultad de Ciencias Exactas, Físico-Químicas y Naturales, Universidad Nacional de Río Cuarto, Ruta Nacional 36, Km 601, Córdoba X5804BYA, Argentina; mfernandez@exa.unrc.edu.ar (M.F.); npaulucci@exa.unrc.edu.ar (N.S.P.); 2 Instituto de Biotecnología Ambiental y Salud, CONICET, Río Cuarto 5800, Argentina

**Keywords:** *Bradyrhizobium* sp., immobilization, *Arachis hypogaea*, cell integrity, long-term storage, PGPR properties

## Abstract

Currently, bacterial inoculant technology focuses on improving long-term storage conditions to ensure adequate rhizobia numbers and their effectiveness as plant growth promoters. This study aimed to investigate whether storage at 4 °C for four years of alginate beads immobilizing *Bradyrhizobium* sp. SEMIA6144 maintains bacterial vitality, efficacy in growth promotion, and ability to establish early interactions with *Arachis hypogaea* L. The recovery of viable SEMIA6144 cells decreased over time (10% at six months, 1% at one year, and 0.01% at four years), while cell vitality remained high at 94.1%, 90.2%, and 93.4%, respectively. The unsaturated/saturated fatty acid ratio declined during storage, reducing membrane fluidity and metabolic activity. Mobility and root adhesion of SEMIA6144 decreased after one and four years. However, growth promotion in peanuts inoculated with SEMIA6144 beads was observed through increased biomass, total chlorophyll, leaf number, leaf area, and decreased chlorophyll fluorescence compared to non-inoculated plants. Although nodulation was low in plants inoculated with four-year-old beads, leghemoglobin levels were maintained. These results demonstrate that *Bradyrhizobium* sp. SEMIA6144 can be stored for four years in alginate beads at 4 °C, maintaining its vitality and ability to establish a symbiosis that stimulates early peanut growth. Understanding these physiological changes could be valuable for the future improvement of long-lasting inoculants.

## 1. Introduction

In recent years, there has been a global drive to develop sustainable technologies to increase crop yields and nutritional value. One of the most important contributions of biotechnology and microbiology to modern agriculture is the use of plant growth-promoting rhizobacteria (PGPR), which manages to decrease the amount of soil chemicals and synthetic fertilizers without affecting crop productivity [1,2]. The survival of rhizobial-based inoculants in the soil is influenced by environmental factors leading to desiccation, exposure of macromolecules to toxic O_2_ levels and high temperatures, and mobilization of inhibitory substances from the seed coat. These factors create a complex environment in which survival responses are difficult to overcome [3]. Inoculant formulations must provide a suitable microenvironment for microbial strains to avoid a rapid decline in cell viability during storage and constitute a source of live cells available in the rhizosphere to interact with plants and the soil microbiome [4,5,6]. A threshold number of cells is essential to obtain the intended positive plant response, for example, 10^6^–10^7^ cells.plant^−1^ for rhizobia. However, in liquid formulations, the introduction of rhizobial suspensions into the soil often results in a rapid decline in bacterial populations [3]. This decline is primarily due to soil heterogeneity, which presents a significant challenge as the introduced bacteria must compete with native microflora that are often better adapted to the environment and withstand predation from soil microfauna [5]. Considering the concept of vitality, which includes a cell’s ability to perform various metabolic, physiological, and genetic functions while maintaining structural and morphological integrity [7], it becomes evident that fluctuating environmental conditions negatively impact bacterial vitality, physiological state, and the establishment of a sufficiently large bacterial population in the rhizosphere, potentially leading to a reduced capacity to provide benefits to crops [8,9]. Therefore, innovative technologies are essential to improve soil delivery and the performance of PGPR formulations. Bioencapsulation emerges to provide a more suitable microenvironment for microorganisms, offering physical protection for an extended period to prevent a rapid decline in introduced bacteria [5]. It facilitates the controlled release of microorganisms into the soil, enhances cell viability, and promotes contact between microbial cells and plants [10,11]. Among the polymers used, alginate is the most widely used since it is a non-toxic, biocompatible, inexpensive, and readily available material [12]. Alginate beads can be used wet or dry in vegetable crop applications [13]. However, in the case of dry beads, cell mortality during the drying of encapsulated cells has been recognized as a critical point in the encapsulation process for non-spore-forming Gram-negative bacteria, which corresponds to most species among PGPR commonly employed as inoculants to establish symbiosis with legumes [5,8,14]. Also, several studies have documented the use of wet alginate bead applications to enhance the growth of various crops of interest, including chili, tomato, wheat, chickpea, and soybean [10,15,16,17,18].

Developing encapsulation methods that enhance cell stability for long periods is essential, as this would significantly contribute to the advancement of inoculant development for legumes. Most solid-based bioinoculants have been reported to have an average shelf life of about 180 days, while liquid formulations can extend this time of commercial use up to two years [5,19]. After that, the number of rhizobia in the inoculants decreases and individual cells may lose efficacy. The ability to store rhizobia inoculants for long periods is essential for their practical use in agriculture, as it ensures the inoculants’ viability and effectiveness when applied to the soil and facilitates their distribution systems, thus providing continuous N fixation benefits and reducing the frequent replacements, offering both economic and environmental advantages [20,21]. Storage temperature is another crucial factor in the survival of immobilized bacteria, along with culture purity and moisture loss during storage [19,22]. Some studies suggest that metabolic changes in immobilized cells result from decreased water activity, oxygen supply, and mass transfer limitations, which alter the microenvironment of these cells [5,23,24]. To survive under varying conditions, bacteria modify their cell membranes to maintain fluidity and essential lipid properties, a process known as homeoviscous adaptation (HA) [25,26]. Recent examples of this adaptive process have been observed in several Gram-negative bacteria, including *Bradyrhizobium* sp. SEMIA6144 underwater deficit [27] and the effects of cold on *Sinorhizobium meliloti* cell membranes [25]. However, there is limited information on how entrapped rhizobacteria adapt their membranes during cold storage and how carriers influence this adaptation.

Legumes are important sources of oil, fiber, micronutrients, minerals, and vegetable proteins suitable for livestock feed and human consumption [28]. Peanut, *Arachis hypogaea*, provides 22% of the plant proteins for human consumption [29]. In Córdoba, Argentina, peanut cultivation faces challenges in its normal physiological development due to unfavorable environmental conditions, such as low soil temperatures (below 18 °C) at the time of planting and N deficiencies in the soils, which adversely affect agricultural productivity [30]. Applications of inoculants based on *Bradyrhizobium* sp. have been widely recommended to inoculate peanuts to stimulate establishment and initial growth, improve biological N fixation, host tolerance to abiotic stress, and increase yields [31,32,33,34,35]. Furthermore, rhizobia inoculation reduces the need for chemical nitrogen fertilizers, thus mitigating the negative environmental impacts of excessive fertilizer use, and also improves soil quality by increasing soil nitrogen content, organic matter, and the formation of stable soil aggregates [36]. Our group has characterized the ability of *Bradyrhizobium* sp. SEMIA6144 to improve peanut plant growth under stress conditions by supplying nitrogen (N) [37,38]. N is a critical limiting element for growth in most plants, constituting an essential component of chlorophyll molecules and playing a vital role in photosynthesis [39,40]. The potential for increasing crop yields by enhancing the photosynthetic rate has been frequently considered, as all dry matter produced is entirely dependent on this process [41]. Peanuts are a C3 crop with high potential photosynthetic capacity; exploring this potential is an effective strategy to improve productivity and achieve yield targets [39].

This research aimed to investigate the preservation of bacterial vitality in *Bradyrhizobium* sp. SEMIA6144 alginate wet beads stored at 4 °C for four years, as well as their efficacy in promoting growth and establishing early interactions with *Arachis hypogaea* L. Elucidating the mechanisms that enable the survival of rhizobia entrapped in alginate and stored at 4 °C for extended periods, along with their physiological adaptability and symbiotic interaction with peanuts, is crucial for validating the utility of immobilization and the performance of the support for microbial inoculation in the soil-plant system.

## 2. Results

### 2.1. Recovery of SEMIA6144 from Alginate Beads After Four Years of Storage at 4 °C

**Figure 1:**
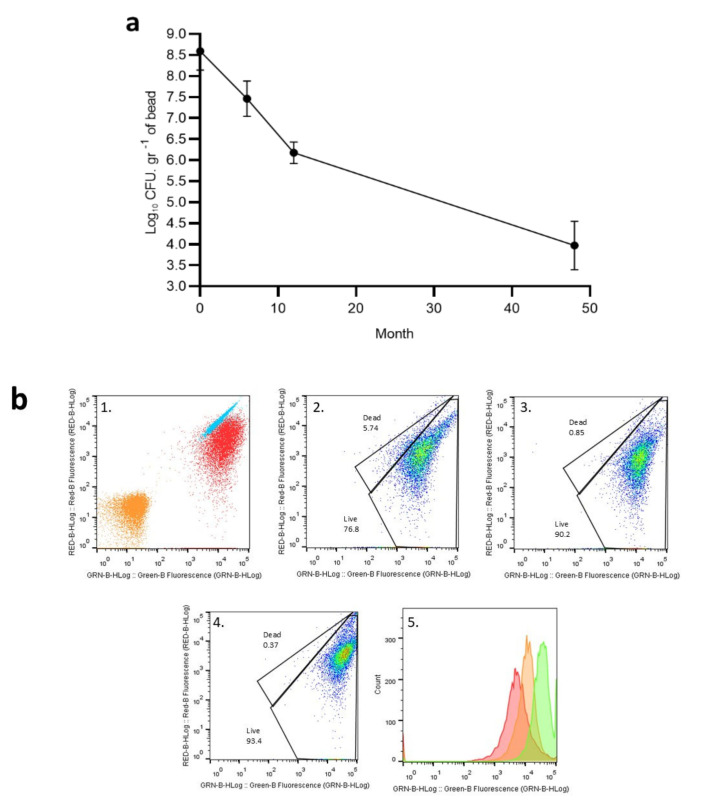
Recovery of *B.* sp. SEMIA6144 cells from alginate beads during storage at 4 °C (**a**); multiparametric graphs obtained by flow cytometry (**b**). The x-axis indicates the log green fluorescence intensity of SYTO9, and the y-axis indicates the log red fluorescence intensity of PI (both log scales). 1. Untreated bacterial controls: (red subpopulation of viable cells with an intact plasma membrane, (blue) subpopulation of dead cells with irreversibly damaged membranes, and (orange) unstained cells; 2. new beads; 3. Six-month beads; one-year-old beads; 4. Four-year-old beads; 5. histogram based on green fluorescence intensity of SYTO9: red: new beads; orange: one-year-old beads; and green: four-year-old beads.

SEMIA6144 demonstrated the ability to survive within alginate beads during four years of storage at 4 °C; however, estimates of the cell population within the beads indicated a decline in bacterial viability over time (Figure 1a). At the time of entrapment, the bacterial population was measured at 10^8^ CFU.gr^−1^, remaining above 10^6^ cfu.gr^−1^ for the first year of storage but subsequently declining to 10^4^ CFU.gr^−1^ per bead after four years.

FCM was first tested on pure cultures of boiled (dead) SEMIA6144 and live (untreated) cells. Live and dead bacterial cells were mixed in different proportions, and the discrimination between live and dead rhizobacteria was positive (Figure 1b, first quadrant). The FCM results observed in Figure 1b (second quadrant) show that approximately 5.7% of the cells in the new beads had a compromised membrane and 77% of live cells. This may be because, now of encapsulation, a late exponential phase culture is used, where all the cells are entrapped, the live and the damaged ones. However, at longer storage time, the percentage of damaged cells inside the bead decreases, leaving only live cells at 94.1%, 90.2%, and 93.4% for the six-month-old and one- and four-year-old beads. As shown in the histogram, the median values for the treatments were 4914, 10,554, and 32,124 for bacteria extracted from new, one-year-old, and four-year-old beads, respectively (Figure 1b, third and fourth quadrant).

### 2.2. Changes in the FA Profile in Entrapped Cells for Long Periods Are Not Enough to Restore Cell Fluidity

**Figure 2:**
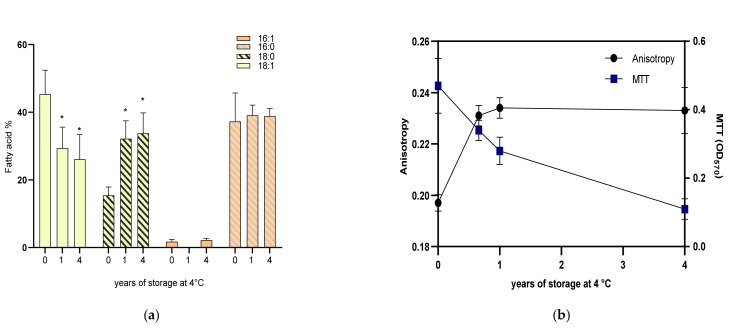
Fatty acid composition of *B.* sp. SEMIA6144 inside the bead of different storage periods at 4 °C (**a**). Asterisks indicate statistical differences for each fatty acid with respect to the new bead (0 year of storage). Microfluidity is informed as polarized values of DPH and metabolic activity as MTT (OD 570) (**b**). Values represent the mean ± SEM of three independent experiments. * indicates a statistically significant difference (*p* < 0.05) for each condition studied with respect to new beads.

The fatty acid (FA) composition of the cell membrane of SEMIA6144 is shown in Figure 2a. The main FA are *cis*-vaccenic acid (18:1^Δ11^) with 73.2%, followed by palmitic acid (16:0) with 17.3%, stearic acid (18:0) with 4.9%, and palmitoleic acid (16:1^Δ9^) with 2.6%. When the microorganism was entrapped in the new bead, the FA profile changed; unsaturated FA (UFA) 18:1 and 16:1 decreased by 38% and 32%, respectively, while saturated FA (SFA) 18:0 and 16:0 increased by 315% and 215%, respectively. As the storage time of the bead at 4 °C increases, such a trend of increasing SFA and decreasing UFA increases for the one- and four-year-old beads concerning the new bead. These changes mainly affected the degree of unsaturation, changing the UFA/SFA ratio (sum of UFA to sum of SFA) from 3.4 to 0.9 for liquid culture and the recently entrapped cells, respectively, reaching a UFA/SFA ratio value of 0.4 for one- and four-year-old beads.

To test the HA of SEMIA6144 to the effects of entrapment and storage at 4 °C on its fluidity, the fluorescence polarization (*P*) of the DPH probe (*P*DPH) was measured. We found that the *P*DPH was close to 0.20 for the liquid culture cells as well as for the recently trapped bacteria, while the *P*DPH increased as the storage time of the beads increased. After six months of storage, *P*DPH was 0.23, remaining at that value until four years of storage (Figure 2b).

When we evaluated metabolic activity by reduction of MTT by cell membrane dehydrogenases, we observed that metabolic activity was reduced by 39% when SEMIA6144 was entrapped in a bead without nutrients compared to bacteria grown in a YEM medium. This activity further declined with storage time, showing decreases of 40% and 76% for one- and four-year-old beads, respectively, compared to the newly entrapped beads (Figure 2b).

### 2.3. SEMIA6144 Can Migrate from the Bead and Attach to Peanut Roots

When we placed beads of different storage periods on mobility plates, we observed that the bacteria diffused from the bead to the agar, indicating that this condition did not affect their motility. Table 1 shows the halo diameter of the swimming motility (swi) of SEMIA6144 from new beads, one and four-year-old beads. The mobility halo diameter was 1.3 cm for both new and one-year-old beads; however, mobility was reduced by 24% for bacteria from four-year-old beads. When a RE-embedded disk was placed on the motility plates, the halo diameter increased by 43%, 38%, and 83% for bacteria from new, one-year-old, and four-year-old beads, respectively.

The attachment and colonization of bacteria on peanut roots are critical steps preceding the initiation of nitrogen-fixing symbiosis. Table 1 shows the Log CFU·mg^−^¹ RD (dry root) of SEMIA6144 cells extracted from beads stored at 4 °C for different periods, adhered to 7-day-old peanut lateral roots. Bacteria from beads of all storage times exhibited adherence to peanut roots; however, the adhesion efficiency of SEMIA6144 decreased with increasing storage duration. Bacteria from one-year-old and four-year-old beads showed a reduction in root attachment by 17% and 49%, respectively, compared to bacteria from newly prepared beads.

### 2.4. SEMIA6144 Retains Peanut Growth Promotion in the Alginate-Based Formulation During Long-Term Storage

We investigated the effect of inoculation with new beads, one- and four-year-old beads, on growth parameters in 30-day-old *A. hypogaea* (Table 2). Inoculation with new beads significantly increased shoot length by 18.2% compared to uninoculated plants. Additionally, inoculation with one- and four-year-old beads resulted in shoot length increases of 29% and 17%, respectively, compared to uninoculated plants, with no statistically significant differences compared to new beads (Table 2). Regarding root length, no statistically significant differences were observed between treatments. Inoculation with new, one- and four-year-old beads significantly increased shoot dry weight by 17%, 25%, and 29%, respectively, compared to uninoculated plants (Table 2), while root dry weight did not significantly increase.

The nodulating symbiosis between SEMIA6144 and peanut remained effective throughout the storage periods studied. Plants inoculated with newly prepared beads exhibited the highest number of nodules (34) and nodular biomass (426 mg) at 30 days of growth. In contrast, plants inoculated with one- and four-year-old beads had 56% fewer nodules and, consequently, lower nodular biomass compared to those inoculated with new beads (Table 2). Regarding the spatial distribution of nodules in the root, in plants inoculated with new beads, 50% of nodules were found in the main root and 50% in the lateral root, while for plants treated with one-year-old beads, 45% of nodules were in the main root and 55% in the lateral roots. For those inoculated with four-year-old beads, 43% were found in the main root and 56% in the lateral root. Interestingly, the leghemoglobin content per gram of fresh nodule was similar across all treatments of plants inoculated with new beads, one-year-old and four-year-old beads (10.2, 11.2, and 12.4 mg·g^−1^ of leghemoglobin per nodule, respectively).

**Figure 3:**
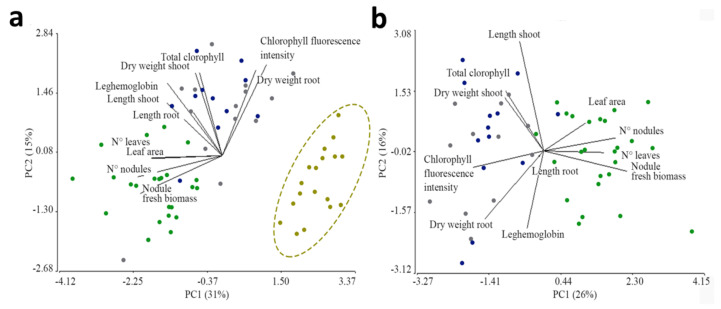
Principal component analysis (PCA) was performed on *A. hypogaea* growth parameters (black lines) and the different inoculation treatments (colored dots): green: new beads; blue: one-year-old beads and gray: four-year-old beads (**a**); mustard: uninoculated, the same treatments except uninoculated treatment (**b**).

In summary, the first two components of the PCA analysis explained 31 and 15% of the total variance observed (Figure 3a). In non-inoculated plants (grouped with mustard ellipse), most of the measured parameters, such as dry biomass, length, number of nodules, nodular biomass, and leghemoglobin content in nodules, showed low values, while plants inoculated with new beads were characterized by a “good state” of their aerial part (given by the number and area of leaves) as well as of their nodules (number and fresh biomass) (Figure 3b). On the other hand, plants inoculated with one- and four-year-old beads showed intermediate values of the parameters measured, with the dry weight of roots and the intensity of chlorophyll fluorescence standing out. In addition, this last parameter was also elevated in uninoculated plants.

### 2.5. SEMIA6144 Retains Beneficial Effects on the Photosynthetic Physiology of Peanuts During Long-Term Storage

**Figure 4:**
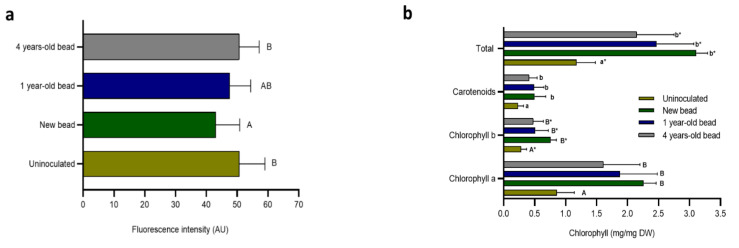

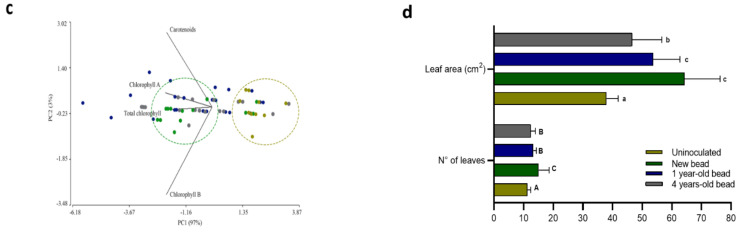
Leaf parameters of 33-day-old *A. hypogaea*. Chlorophyll fluorescence intensity (arbitrary units) (**a**); Chlorophyll concentration in leaf discs of peanut plants (**b**); principal component analysis (PCA) based on the photosynthetic parameters (black lines) and the different inoculation treatments in plants: uninoculated, new, one- and four-year-old beads (brown, green, blue, and gray dots, respectively). * indicates a statistically significant difference (*p* < 0.05). Physicochemical parameters included: total chlorophyll, chlorophyll a, chlorophyll b, and carotenoids (**c**); (**d**) leaf area and number of leaves per peanut plant (**d**). Different letters indicate a significant difference between treatments in each column for each parameter evaluated (*p* < 0.05).

The fluorescence intensity of 30-day-old peanut leaves of uninoculated and inoculated with beads of different storage periods is shown in Figure 4. It can be observed that, in uninoculated plants and those inoculated with four-year-old beads, the chlorophyll fluorescence intensity was 15% higher compared to the leaves of plants inoculated with new beads (Figure 4a). If our assumption is valid, we can infer an inverse relationship between observable fluorescence and photosynthetic efficiency, suggesting that the presence of an appropriate inoculum of SEMIA6144 improves the photosynthetic efficiency of peanut, as evidenced by the significant decrease in chlorophyll fluorescence intensity in plants inoculated with new beads. However, correlation analysis between chlorophyll fluorescence and chlorophyll content revealed no significant correlation for any of the treatments studied (*p* > 0.05; correlation coefficient close to zero; for more details, see Table S1, Supplementary Materials).

Total chlorophyll content increased significantly in plants inoculated with new beads (163%), as well as in those inoculated with one-year-old beads (109%) and four-year-old beads (82%) compared to uninoculated plants. In all cases, chlorophyll a was the predominant form. The number of carotenoids, which are associated with plant protection and physiological health, also increased in the treatments with newly prepared beads, one- and four-year-old beads, by 117% and 78%, respectively, compared to uninoculated plants (Figure 4b). The first two components of the PCA analysis explained 97% and 3% of the total observed variance, respectively (Figure 4c).

All photosynthetic physiology parameters, including carotenoids and chlorophyll (a, b, total), were displayed, and the samples were distributed along the X-axis according to the storage duration of the beads. Samples from plants inoculated with new beads, which contained higher concentrations of photosynthetic parameters, were primarily located on the right side of the graph, while samples from plants inoculated with beads exhibiting lower values for these parameters were positioned on the left side. In contrast, samples from plants inoculated with one- and four-year-old beads were more evenly distributed between both groups.

The correlation analysis between the variables related to the total chlorophyll, chlorophyll a, b, and carotenoids content indicated that for the new, one-year-old and four-year-old beads, a positive correlation was observed between all the pigments when compared in pairs (*p* < 0.05, ~0.96–1.00 correlation coefficient). While for uninoculated only a positive correlation was observed between Chl a and b and total chlorophyll with both Chlo a and Chlo b (*p* < 0.05, ~0.99–1.00 correlation coefficient). For more details, see Table S2, Supplementary Materials.

The results presented in Figure 4d indicate that inoculation with SEMIA6144 beads significantly increased the number of leaves per plant by 34%, 17%, and 10% for plants inoculated with new beads, one- and four-year-old beads, respectively, compared to uninoculated plants. Additionally, inoculation with SEMIA6144 beads of different storage durations had significant effects on leaf area in comparison to uninoculated plants. Inoculated peanuts exhibited significant increases in leaf area of 69%, 39%, and 22% for treatments with new beads, one- and four-year-old beads, respectively, compared to uninoculated plants (Figure 4d).

## 3. Discussion

Bacterial viability currently refers to the ability of a cell to grow and reproduce under specific environmental conditions [42]. Several authors have demonstrated that encapsulation significantly improves the survival of microorganisms compared to free microorganisms [43,44,45]. In our study, over a period of four years of storage at 4 °C, the percentage recovery of viable SEMIA6144 cells inside the beads was 10% at six months, 1% at one year, and 0.01% after four years at 4 °C, compared to the initial number of immobilized cells. Similar results were reported by [46], who described that the *Bacillus subtilis* and *Pseudomonas corrugata* population in alginate beads was 0.01% (10^6^ CFU.gr^−1^) concerning the initial number of immobilized cells after three years. Viability is usually associated with the ability to reproduce and, subsequently, with culturability. However, not all bacterial cells conform to this relationship; even if they cannot reproduce and grow under specific conditions, they may retain many properties of viable, fully functional cells, thus being considered cells with vitality [7]. In our work, FCM outlined the vitality of SEMIA6144 immobilized in alginate beads and stored for four years at 4 °C by dual SYTO9/PI staining. Interestingly, our results reveal for the first time that, the longer the storage time, the vitality of SEMIA6144 cells immobilized inside the bead was 94.1%, 90.2%, and 93.4% for six months, one- and four-year-old beads, respectively, while the percentages of damaged cells inside the bead decrease (lower percentage of dead cells) with storage time. From a biotechnological perspective, it is crucial to have a lower number of dead cells inside the beads, as they lack the minimum structural integrity and ability to carry out basic cellular functions, lacking both vitality and viability [47]. The encapsulation of microorganisms in an alginate matrix isolates them from environmental influences that may reduce their survival [48]. Additionally, as a permeable polymer, alginate facilitates the exchange of nutrients in the soil, promoting the survival and replication capacity of microorganisms. The survival of encapsulated microorganisms depends on factors such as the initial concentration and metabolic state of the inoculum, the alginate concentration, and the methods of storage and capsule preparation [49]. It has been demonstrated that an alginate concentration of 2% positively affects the survival of microorganisms compared to lower concentrations, particularly when exposed to elevated temperatures [50]. Furthermore, lower storage temperatures have been observed to favor higher survival rates of microorganisms, as lower temperatures are more conducive to preserving microbial activity within the capsules [49]. Ref. [51] analyzed the effect of temperature and found that samples of alginate-perlite encapsulated *Pseudomonas putida* stored at 4 °C in a 0.9% NaCl solution showed no surface damage or capsule leakage, while those maintained at room temperature exhibited a slight decrease in microbial content. A similar trend was observed by [52], who reported a 100% survival rate at both temperatures of microbial fertilizer based on clay and alginate encapsulation.

The bacterial lipid envelope is essential for the survival of microorganisms exposed to external physical and chemical factors [53] and greatly influences the process of symbiotic bacteria-plant interaction [54]. Phosphatidylcholine is a phospholipid present in the membranes of bacteria of the *Rhizobiaceae* family that usually has palmitic acid and oleic or vaccenic acid as majority fatty acids and is essential for symbiotic interactions of rhizobia with legumes [25,27,55,56,57,58]. It is of vital importance that bacteria maintain their membrane fluidity at optimal values to ensure physiological homeostasis and the integrity of all processes taking place in their membranes [25]. Generally, the ratio of UFA to SFA in the bacterial cell membrane is used as an indirect indicator of membrane fluidity, as a higher UFA/SFA ratio is usually associated with higher fluidity [59]. Our experimental results show a correlation between the UFA/SFA ratio and SEMIA6144 membrane fluidity. During storage of the beads at 4 °C, the UFA/SFA ratio decreased due to a significant increase in 18:0 and 16:0 SFA, leading to a reduction in membrane fluidity. When exposed to environmental stressors, such as extreme temperatures or nutrient shortages, bacteria may adjust their fatty acid composition to improve their survival. For example, an increase in SFA helps stabilize the membrane under these storage conditions (4 °C) [60,61], but this can also slow down metabolism as a means of conserving energy [62]. Ref. [25] showed that when *Sinorhizobium meliloti* is subjected to thermal changes, variations in fatty acid composition and acyl chain shortening occur to compensate for temperature-induced changes in membrane microviscosity. However, in our work, it was observed that despite the increase in saturated fatty acids as well as acyl chain shortening in the membrane of SEMIA6144 immobilized for four years, it did not restore the initial fluidity of the membrane. Based on these results, we can suggest that the inability to maintain membrane fluidity for prolonged periods may be related to the reduced metabolic activity of SEMIA6144 inside the beads. In addition, inadequate control of membrane fluidity could be related to problems interacting with the host plant. It has been reported that reduced fluidity may also limit the functionality of membrane proteins involved in symbiosis, such as those that facilitate signal exchange with the host plant and nodule formation [63]. In summary, an increase in saturated fatty acids in response to elevated temperatures helps to maintain membrane stability. However, it may affect the dynamics of the interaction with the plant, resulting in a less efficient symbiosis compared to the new beads.

When bacteria compete for the same resources, chemotaxis toward exuded compounds becomes a fundamental trait for root colonization and plant-facilitated microorganism selection [64,65]. Our working group previously described the peanut RE positive effect on cell motility and adhesion of SEMIA6144 immobilized alginate beads to peanut roots [37]. In the current work, we have demonstrated the ability of immobilized SEMIA6144 to leave the beads by swimming-like mobility. However, we observed a 7.5% and 24% reduction in motility for immobilized SEMIA6144 after one and four years, respectively. Notably, the addition of RE disc to the culture medium surprisingly enhanced the motility of SEMIA6144, irrespective of the storage period of the beads. These results suggest that SEMIA6144, immobilized in alginate beads and stored for four years at 4 °C, can migrate from the beads to the rhizosphere when stimulated by the presence of RE. These results could be associated with molecules exuded by peanut, mainly involved in legume-rhizobium interactions, as high concentrations of the flavonoid lutein related to a positive chemotaxis of SEMIA6144 towards RE and other chemoattractants such as malic acid and aromatic compounds increase the swimming speed of various PGPR strains [37,38,66,67]. Similar to previous work reported for immobilized SEMIA6144 and *Azospirillum brasiliense* Az39 [37], our research confirms that with increasing storage time, the attachment decreases. Specifically, we observed reductions of 16% and 49% in attachment capacity for SEMIA6144 immobilized and stored for one year and four years at 4 °C, respectively. This reduction may be associated with decreased swimming motility. [68] demonstrated that flagellar alterations can lead to a complete loss of swimming ability, increased biofilm formation, and reduced bacterial attachment to root hairs. However, further studies are required to determine the reasons why swimming and root bacterial attachment decrease with storage time.

To validate its use as a long-life inoculant, it is crucial to assess the persistence of the PGPR properties of *Bradyrhizobium* sp. SEMIA 6144 after immobilization in alginate beads and storage for a period of four years. The results obtained in this study provide a solid foundation for the continued development of our research, as reported in [37]. Growth promotion of peanut plants was evidenced mainly in the aerial part, with significant improvements in biomass, total chlorophyll, and decreased chlorophyll intensity by inoculation with SEMIA6144 beads compared to non-inoculated. This improvement in peanut growth parameters by SEMIA6144 could be directly related to its ability to fix atmospheric N in the nodules and provide it to the plant in the form of ammonium. Previously, we demonstrated that inoculation of peanuts with SEMIA6144 significantly improves N content in leaves (82%) compared to uninoculated plants [38].

Early seedling emergence and growth are important characteristics for obtaining good health, plant density, and high subsequent yields, particularly in regions where low soil temperatures prevail at the time of sowing, such as in the Cordoba region [33,34,69]. Numerous studies have reported significant beneficial effects of immobilized bacterial inoculants on plant growth and productivity under laboratory, greenhouse, and field conditions [70,71]. Like our results, ref. [10] reported wheat growth promotion by increasing the aerial part when inoculated with *P. fluerescens* wet beads compared to the control treatment (autoclaved beads). The improvements observed in our work could be attributed to an enhanced biological fixation of nitrogen by the inoculant. Many authors, as well as our previous work, show that the appearance of nodules in peanuts was recorded after 12 and 20 days and that the nitrogenase activity was initiated after 11 days, which was irrespective of the presence of mineral N [38,72]. In this study, we observed that in plants inoculated with four-year-old beads, the number and biomass of nodules per plant decreased. We found no evidence that rhizobia in older beads (i.e., older than one year) had lost the ability to grow on nutrient agar, survive in the beads, and nodulate and fix nitrogen with the host plant. However, we can suggest that the reduction in numbers and nodular biomass may be related directly to the lower availability and basal metabolic state of bacteria in the four-year-old beads compared to fresh beads, as a high number of bacteria in the soil is essential for ensuring nodule formation in legumes. Like our data, ref. [73] showed that in legume inoculants over 18 months of storage, there is a slight decrease in nodulation capacity due to reduced viability of rhizobia over time. However, the decrease is not significant enough to impair the growth promotion efficiency of the plants, even after 18 months of storage. Furthermore, the reduction in bacterial motility and adhesion to roots under these conditions are factors that impact nodulation efficiency and competitiveness [74,75]. However, the levels of leghemoglobin in nodules from plants inoculated with four-year-old beads were comparable to those observed in nodules from plants inoculated with new beads, suggesting optimal symbiotic nitrogen fixation [76]. Ref. [7] indicated that the presence of leghemoglobin is essential for nitrogenase enzyme activity, emphasizing the relationship between leghemoglobin content and nitrogenase activation.

Our results also show that inoculation of peanuts with beads of different storage times increased the aerial biomass by approximately 25% compared to uninoculated plants. Several studies show that different strains of *Bradyrhizobium* SEMIA6461 and SEMIA6462 contribute significantly to the production of *Vigna unguiculata*, producing 21% more dry matter of the aerial portion than the non-inoculated treatment under greenhouse and field conditions [77,78,79]. The increase in nitrogen availability through fixation by symbiotic bacteria, such as *Bradyrhizobium*, promotes the synthesis of amino acids, proteins, and chlorophyll, leading to improved growth and greater biomass accumulation in stems and leaves [80]. The improvement in photosynthetic activity is also reflected in parameters related to chlorophyll fluorescence [76]. In our study, plants inoculated with SEMIA6144 beads, regardless of storage time, increased levels of total chlorophyll content in leaves while decreasing chlorophyll fluorescence intensity compared to uninoculated plants. Some authors have described that in healthy plants, up to 80% of the absorbed energy is transferred under stationary conditions to the photochemical pathway; only 2–5% represents chlorophyll fluorescence, and the rest is dissipated as heat [81]. Thus, the inverse relationship between photochemical and non-photochemical processes (heat dissipation, chlorophyll fluorescence) can be seen in our work, indicating that inoculation with SEMIA6144 beads could favor the photosynthetic process. The increase in photosynthetic rate is beneficial to the plant; in soybean (*Glycine max* L.) and pea (*Pisum sativum* L.), high-yielding genotypes also showed a high photosynthetic rate [82,83].

About photosynthetic pigments, our experiment demonstrated that the carotenoid content in the leaves of 30-day-old peanut plants increased following inoculation with SEMIA6144 new, one- and four-year-old beads. This could be a factor why inoculation of peanuts with SEMIA6144 immobilized on alginate beads improved photosynthetic characteristics over uninoculated ones because carotenoids are efficient antioxidants and, therefore, higher carotenoid content in plants help to maintain higher chlorophyll content by protecting the chloroplast [84,85]. Similarly, ref. [86] reported an increase in chl a and carotenoid content in tomato plants inoculated with wet *M. oryzae* chitosan beads concerning inoculation with free cells. Chlorophyll measurements could be a reliable method to examine the efficiency of rhizobia trapped and stored for long periods [87]. There are few reports in the literature on the PGPR effects of inoculating plants with beads stored for long periods. We have identified a negative correlation between the reduction in chlorophyll concentration, leaf area, and leaf count as a function of bead storage time. The decrease in the total number of nodules, highlighted in plants inoculated with beads stored for 4 years, could be a determining factor in the decrease of the characteristics associated with photosynthesis.

## 4. Material and Methods

### 4.1. Encapsulation of Bradyrhizobium sp. SEMIA6144 in Alginate Beads

*Bradyrhizobium* sp. SEMIA6144 (referred to as SEMIA6144 in the rest of the text) was cultivated as described by [37]. The bacterial biomass obtained after centrifugation at 10,000 rpm for 10 min was resuspended in a sterile physiological solution (0.9% *w*/*v* NaCl). For encapsulation, a 20% *w*/*v* sodium alginate solution (Sigma-Aldrich, St. Louis, MO, USA) was prepared in water at 45 °C and sterilized. The sodium alginate solution was then added to the bacterial suspension (2% *v*/*v*) and mixed at 30 °C for 30 min. Beads were obtained using the ionic gelation method [88], where the mixture was dripped into a 2% CaCl_2_ solution and stirred for 30 min. The beads were separated from the suspension, washed three times with 500 mL of saline, and stored aseptically in vials at 4 °C for four years. For all subsequent determinations, 1 g of beads was dissolved in 3 mL of citrate buffer (pH 7) (33.3% *w*/*v*) in a test tube for 30 min at room temperature [37].

### 4.2. Count of Viable Bacteria in Beads from Long-Term Storage

First, the stored beads to be used were checked for contamination. The bacterial count was carried out by the microdroplet technique described by [89] in the YEM medium [90], and plates were incubated at 28 °C for 5 days. The result is expressed as Log 10 CFU.gr^−1^ of beads. These viability tests were performed both at the time of bead manufacture, at six months of storage, at one year, and four years of storage at 4 °C.

### 4.3. LIVE/DEAD BacLight Bacterial Vitality Assay in Combination with Flow Cytometry

To determine the percentages of live and dead cells inside the beads over the 4 years of storage, bacterial staining with the commercial LIVE/DEAD BacLight kit (Molecular Probes^®^, Life Technologies, Carlsbad, CA, USA) was combined with flow cytometry. For this purpose, 1 g of new, one and four-year-old beads were dissolved in 3 mL of citrate buffer for 30 min at room temperature. Subsequently, the bacterial suspension was centrifuged at 12,000 rpm for 15 min, and the biomass was suspended in 100 µL of sterile saline solution. The suspensions obtained were used for live and dead staining. The stock solutions of the dyes were prepared as follows: propidium iodide (PI, 20 µM) and SYTO9 (5 µM) were used as proposed by the manufacturer. Samples obtained from the beads were immediately stained with 0.3 µL of a SYTO9-IP mixture (1:1) and incubated in the dark at room temperature for 15 min, respectively, before analysis [91,92]. The cell samples were washed three times with a physiological solution. Finally, the biomass stained with the fluorophore mixture was resuspended in 100 µL of physiological solution for subsequent analysis by flow cytometry.

Flow cytometric (FCM) measurements were performed on a Guava^®^ easyCyte system (Cytek^®^ Biosciences, Fremont, CA, USA), with excitation at 488 nm. The optical filters were set so that red fluorescence was measured at 695/50 nm (RED-B or FL3) and green fluorescence was measured at 525/30 nm (GRN-B or FL1). Excitation of SYTO9 at 480 nm produces green fluorescence emission at 500 nm, whereas excitation of PI at 490 nm generates red fluorescence above 635 nm [93,94]. Viable cells (with intact membranes) are only permeable to SYTO9 but not to PI; therefore, viable cells emit green fluorescence [95]. In contrast, dead cells (with permeabilized membranes) are stained with PI (it replaces SYTO9 for being more concentrated) and emit red fluorescence.

For instrument adjustment, single and double color controls were used. For this, a stationary-phase culture of SEMIA6144 was used. One aliquot of the culture was not stained with any fluorophore (autofluorescence control), one aliquot was stained with SYTO9 (live cell control), and one of the aliquots of the bacterial suspension was heated at 100 °C for 10 min to permeabilize cell membranes and cause cell death and stained with IP (dead cell control). Once the populations were located through double-color controls (SYTO9: IP mixture) of live and dead cells, we proceeded to analyze the bacterial samples extracted from new, one-year and four-year storage beads stained with the SYTO9: IP mixture. The data obtained in the flow cytometer were analyzed using FlowJo_V10 software.

### 4.4. Metabolic Response of Bacterial Cells to Immobilization and Long-Term Storage at 4 °C

The metabolic activity of cells immobilized on new, one- and four-year storage beads was assessed by the 3-(4,5-dimethylthiazol-2-yl)-2,5-diphenyltetrazolium bromide (MTT) colorimetric assay [96] as described in [37]. The absorbance was at 560 nm using an ELISA reader plate (Thermo Scientific, Waltham, MA, USA, Multiskan FC).

### 4.5. SEMIA6144 Fatty Acid Composition and Membrane Fluidity

The biomass obtained from 1 g of new, one- and four-year-old storage beads was washed and resuspended in 600 μL of physiological solution, and from there, lipid extraction, methylation, GC analysis, and anisotropy. In addition, both determinations were compared with an aliquot of the biomass of a SEMIA 6144 culture in the stationary phase.

#### 4.5.1. Lipid Extraction and Analysis of Fatty Acids by GC

Total lipids from bacteria immobilized on new, one-year, and four-year storage beads were extracted following the protocol described by [97]. The lower lipid-containing phase was dried under and dissolved in 1 mL of boron trifluoride (methanol 20%) and heated at 100 °C for 2 h to obtain the methyl esters of fatty acids (FAMEs). FAMEs from SEMIA6144 cells were analyzed using a 5890 II gas chromatograph (Hewlett-Packard, Palo Alto, CA, USA) equipped with a highly polar column, HP 88, and a flame ionization detector was used. Gas chromatograph conditions were descripted by [37]. FAMEs were identified by comparing retention times to commercial standards (Sigma Chemical Co., St. Louis, MO, USA).

#### 4.5.2. Determination of Membrane Microfluidity

The fluidity of membrane cells was determined by measuring the fluorescence polarization of the 1,6-diphenyl-1,3,5-hexatriene (DPH) probe (Invitrogen, Waltham, MA, USA) inserted into the membrane. Following the procedures described by [98], SEMIA6144 cells extracted from alginate beads from different storage periods were washed in a sterile physiological solution and resuspended in the same solution to 0.2 OD at 600 nm. Then, 1 µL of fluorescent probe (stock solution diluted to 12 mmol in tetrahydrofuran) was added to 3 mL aliquots of resuspended cells to give a final probe concentration of 4 mmol. The aliquots were incubated with a magnetic stirrer at 200 rpm for 10 min in the dark at room temperature.

Fluorescence intensity (FI) measurements were performed using a FluoroMax R-Spex 4 Jovin Yvon (Horiba, Piscataway, NJ, USA) spectrofluorometer equipped with excitation and emission polarizers. The widths of the excitation and emission beam slits were set at 5 nm. FI was measured at excitation and emission wavelengths for DPH and set at 358 and 428 nm [99].

### 4.6. Early Interaction Parameters of Immobilized Bacteria with Peanut

Swimming and chemotaxis phenotypes were tested on water agar plates containing 0.3% agar-agar. Two plate formats were used. First, 90 mm Petri dishes were filled with 25 mL of swimming agar. The swimming plates were inoculated by inserting new, one-year-old, and four-year-old storage beads in the center of the plate into the agar. Second, to find out whether the presence of seven-day-old peanut root exudate (RE) modifies bacterial motility, a paper disk soaked with 10 μL of RE was placed on the swimming plates and inoculated as described above. Plates were incubated at 28 °C for 7 days, and the diameter of the motility halo was subsequently measured [100]. Seven-day-old peanut RE were collected, as described in [38]. For SEMIA6144 adhesion to peanut roots, seven-day-old seedlings were selected, and 100 mg of 2-cm-long lateral roots were cut. Furthermore, the cells obtained from 1 g of new, one-year-old, and four-year-old storage beads were washed and resuspended in 1000 μL of physiological solution, and viable cell counts were performed for each suspension. In tubes containing 1000 μL of the bacterial suspension from each bead condition, 100 mg of lateral roots were added and gently shaken during incubation on a rotary shaker at 50 rpm at 28 °C [101]. After 2 h, the bacterial suspension was removed, and the roots were washed 7 times with a physiological solution to remove free and weakly adherent bacteria. Counting the number of adherent bacteria was performed using the microdroplet technique. All experiments were repeated at least twice with two or three test samples.

### 4.7. Growth Parameters of Peanut Plants Inoculated with Beads from Different Storage Times

*Arachis hypogaea* L. seeds were surface sterilized according to the method described by [102], germinated in Petri dishes containing 0.8% water-agar, and subsequently placed in plastic pots with a diameter of 5 cm and a height of 10 cm, filled with sterile vermiculite as the substrate. To evaluate the effect of inoculation with the new, one-year-old, and four-year-old SEMIA6144 beads, 1 g of alginate beads was manually added per seed using a sterile spoon. Additionally, non-inoculated plants served as negative controls. The pots were incubated in a growth chamber at 24 °C under a 16 h light/8 h dark photoperiod [103]. Each treatment was irrigated twice a week, alternating between water and nutrient solution without N source (Hoagland solution). Twelve (12) replicates were used for each treatment.

#### 4.7.1. Morphological and Growth Parameters

At 33 days of growth, the plants were harvested. The length and dry weight of the shoot and root, as well as the number of nodules and leaves of 12 plants of each treatment, were weighed separately (in triplicate). To determine the dry plant biomass, plants were dried to constant weight at 75 °C.

To estimate leaf area, one leaflet from each quadrifoliate leaf was collected from various sections of the plants in each treatment. The selected leaflets were digitized using a scanner at 300 dpi, and their areas were measured with ImageJ^®^ software ver. 1.53t. For accurate measurements, the inoculated leaves were photographed against a contrasting background with a known scale (ruler) to calibrate the software. The photos were processed using ImageJ^®^ software to obtain the leaf area. The average distance between the camera and the leaf was 50 cm. The area of each leaflet was multiplied by four (corresponding to the number of leaflets per quadrifoliate leaf), and the average was calculated and multiplied by the total number of leaves per plant [104]. The experiment was conducted on three plants per treatment with three independent replications.

#### 4.7.2. Leghemoglobin Content in Nodules

After washing the roots with water to remove vermiculite, nodules were harvested, counted, and their fresh weight was recorded. Nodule samples were homogenized in aliquots of Drabkin’s reagent at a 1:1 to 1:2 (*w*/*v*) ratio, and leghemoglobin content was quantified spectrophotometrically at 540 nm, following the method described by [105]. Bovine hemoglobin was used as the standard, and the results are expressed as mg g^−1^ of nodule fresh weight.

#### 4.7.3. Chlorophyll Fluorescence

Chlorophyll fluorescence was measured using an Odyssey CLx Imager (LI-COR Biosciences, Lincoln, NE, USA), equipped with a solid-state diode laser providing light excitation at 685 nm and generating emission images (~700 nm) of fluorescent chlorophyll in the leaves. The instrument was operated using Image Studio Software ver. 6.0. Eight fully expanded leaves were sampled from each treatment, immediately placed in plastic bags, and stored in a refrigerator to prevent exposure to direct light. Fluorescence images from each treatment were analyzed using ImageJ software [106].

#### 4.7.4. Photosynthetic Pigments

Chlorophyll (chl) a, chlorophyll b, and carotenoid (car) concentrations were determined following the method of [107]. Leaf discs (6 mm in diameter) were obtained from each of the four leaflets of the newest, fully expanded, and photosynthetically active leaves. The discs were extracted in 1 mL of 80% (*v*/*v*) acetone in the dark for 48 h at 4 °C. Ten independent samples were collected from each treatment to determine pigment concentrations. The extracts were analyzed spectrophotometrically at 664, 647, and 470 nm using a double-beam UV-visible spectrophotometer (Beckman DU 640, Brea, CA, USA) with 1 cm pathlength glass cuvettes. The solutions were diluted in 80% acetone to achieve absorbance values within the range of 0.1 to 0.7. The concentrations of chlorophyll a, chlorophyll b, carotenoids, and total chlorophyll were calculated using the following equations:

*Chla* = 12.25 · *A_663nm_* − 2.79 · *A_647nm_*

*Chlb* = 21.5 · *A_663nm_* − 5.1 · *A_663nm_*

*Cars* = (1000 × *A_470nm_* – 1.82 × (*Chla*) – 85.02 × (*Chlb*))/198

*Chl Total* = 17.95 · *A_647nm_* · 28 + 7.9 · *A_663nm_*

where *A*646.8 and *A*663.2 are the absorbance at wavelengths 646.8 and 663.2 nm, respectively. Extract concentrations were calculated by multiplying the concentrations of the diluted samples by their respective dilution factors. The weights of chlorophyll a, chlorophyll b, and total chlorophyll (a + b) were determined based on the fresh weight (mg) of each disk used for analysis. Each experiment was conducted in triplicate, and the mean value was reported. Weights are expressed on a wet basis unless otherwise specified.

### 4.8. Statistical Analysis

Results were expressed as means (from three biological replicates) and standard errors. The data were statistically analyzed on the InfoStat Software (version 2018I). For bacteria-related data, one-way analysis of variance (ANOVA) was performed, followed by Tukey’s test as a posteriori test, when necessary, with a significance level of 0.05 (*p* < 0.05). For growth parameters of plants, the means compared with Fisher’s least difference test (*p* < 0.05) were used. Moreover, a Pearson correlation test between different photosynthetic pigments was used, while principal component analysis (PCA) was used to visualize the overall patterns of dispersion among the different samples (uninoculated and inoculated plants: new, one-year-old, and four-year-old beads) and to check correlation with the set of morpho-physiological parameters of plants.

## 5. Conclusions

This study demonstrates that *Bradyrhizobium* sp. SEMIA6144 exhibits survival and vitality inside the beads, even after a storage period of four years at 4 °C. Furthermore, our results indicate that this inoculant, despite exhibiting inhibited metabolic activity during immobilization, significantly enhances the growth and photosynthetic yield of peanuts after four years of storage. However, the four-year-old bead exhibits lower efficiency in improving peanut growth parameters, concerning new beads. Therefore, the efficiency of aged beads could be improved by inoculating the plants with a greater number of beads, increasing the concentration of bacteria per bead, or enriching the beads with nutrients. These findings suggest not only the long-term robustness of this inoculant immobilized on alginate beads but also its promising potential for improving crop performance in terms of growth and the efficiency of the photosynthetic apparatus.

The findings from this research allow us to propose as future perspectives the evaluation of the survival of SEMIA6144 under different storage conditions and its application in field trials.

## Figures and Tables

**Table 1:** Early interaction parameters of entrapped *B*.sp. SEMIA6144 with *A. hypogaea* seedlings.

Early Interaction Parameters	Motility Halo Diameter (cm)		Root Adhesion (Log_10_CFU·mg^−1^ RD)
	Swimming	Swimming + RE	
New bead	1.40 ± 0.35 b	1.95 ± 0.30 a	4.90 ± 0.40 c
1-year-old bead	1.30 ± 0.30 b	1.80 ± 0.20 a	4.10 ± 0.08 b
4-years-old bead	1.00 ± 0.40 a	1.90 ± 0.20 a	2.50 ± 0.10 a

Swimming-type mobility of entrapped bacteria in the presence or absence of disc with radical exudate (RE) and adhesion of bacteria extracted from new beads and from four years of storage to lateral roots of seven-day-old peanut plants. Different letters indicate a significant difference between the treatments in each column for each growth condition (*p* < 0.05).

**Table 2:** Effect on *A. hypogaea* growth variables after inoculation with alginate beads containing *B.* SEMIA6144 after one and four years of storage at 4 °C.

Treatments	Length (cm)		Dry Biomass (mg)		Nodules		
	Shoot	Root	Shoot	Root	N° Nodules	Biomass (mg)	Leghemoglobin (mg.gr^−1^ Nodule)
Uninoculated	18.10 ± 4.0 a	20.9 ± 3.2 a	693.7 ± 209 a	236.5 ± 49 ab	-	-	-
New bead	21.40 ± 2.7 b	24.4 ± 3.4 a	812.0 ± 278 b	181.8 ± 61 a	33.8 ± 6.0 c	426.0 ± 47 c	10.2 ± 1.3 b
1 years-old bead	23.40 ± 2.8 b	25.0 ± 3.8 a	866.0 ± 110 b	239.0 ± 55 b	14.5± 3.4 b	115.0 ± 38 b	11.2 ± 3.4 b
4 years-old bead	21.25 ± 2.6 b	23.0 ± 2.7 a	894.4 ± 186 b	263.7 ± 62 b	15.3± 1.7 b	8804 ± 29 b	12.4 ± 3.7 b

Different letters indicate a significant difference between the treatments in each column for each growth condition (*p* < 0.05).

## Data Availability

Data will be made available upon request.

## Supplementary Materials

The following supporting information can be downloaded at: https://www.mdpi.com/article/10.3390/plants13212983/s1, Table S1. Pearson correlation (coefficient-*p* valor) between the different photosynthetic pigments and fluorescent chlorophyll for the different treatments. Table S2. Pearson correlation (coefficient-p valor) between the different photosynthetic pigments and fluorescent chlorophyll for the different treatments: a (uninoculated), b (new bead), c (one-year-old bead), and d (four-year-old bead).
