# Supplementary material for: Long-Life Inoculant: Bradyrhizobium Stored in Biodegradable Beads for Four Years Shows Optimal Cell Vitality, Interacts with Peanut Roots, and Promotes Early Growth
Source: Plants (Basel). 2024 Oct 25;13(21):2983. doi: 10.3390/plants13212983 (PMC11548396; doi:10.3390/plants13212983)
Supplement: Supplementary file 1 [file plants-13-02983-s001.zip › plants-3262421-supplementary.pdf]

Supplementary material

Table S1. Pearson correlation (coefficient-*p* valor) between the different photosynthetic pigments and fluorescent chlorophyll for the different treatments.

**Uninoculated treatment**	*Fluorescence chlorophyll*	*Chl a*	*Chl b*	*Cars*	*Total chlorophyll*
Fluorescence chlorophyll	1.00-<0.0001	0.04-0.88	0.10-0.72	0.10-0.72	0.07-0.80
**New bead**					
Fluorescence chlorophyll	1.00-<0.0001	0.41-0.11	0.31-0.24	0.34-0.19	0.39-0.13
**1 year-old bead**					
Fluorescence chlorophyll	1.00-<0.0001	-0.55-0.02	-0.54-0.03	-0.63-0.0087	-0.55-0.02
**4 years-old bead**					
Fluorescence chlorophyll	1.00-<0.0001	0.31-0.24	0.33-0.20	0.35-0.19	0.32-0.23

Table S2. Pearson correlation (coefficient-*p* valor) between the different photosynthetic pigments and fluorescent chlorophyll for the different treatments: a (uninoculated), b (new bead), c (1 year-old bead), d (4 years-old bead).

Uninoculated	*Fluorescence chlorophyll*	*Chl a*	*Chl b*	*Cars*	*Total chlorophyll*
Fluorescence chlorophyll	1.00-<0.0001	0.04-0.88	0.10-0.72	0.10-0.72	0.07-0.80
Chl a		1.00-<0.0001	**0.93-<0.0001**	0.50-0.04	**0.99-<0.0001**
Chl b			1.00-<0.0001	0.23-0.38	**0.97-<0.0001**
Cars				1.00-<0.0001	0.41-0.11
Total chlorophyll					1.00-<0.0001
New bead	*Fluorescence chlorophyll*	*Chl a*	*Chl b*	*Cars*	*Total chlorophyll*
Fluorescence chlorophyll	1.00-<0.0001	0.41-0.11	0.31-0.24	0.34-0.19	0.39-0.13
Chl a		1.00-<0.0001	**0.97-<0.0001**	**0.97-<0.0001**	**1.00-<0.0001**
Chl b			1.00-<0.0001	**0.91-<0.0001**	**0.98-<0.0001**
Cars				1.00-<0.0001	**0.96-<0.0001**
Total chlorophyll					1.00-<0.0001
1 year-old bead	*Fluorescence chlorophyll*	*Chl a*	*Chl b*	*Cars*	*Total chlorophyll*
Fluorescence chlorophyll	1.00-<0.0001	-0.55-0.02	-0.54-0.03	-0.63-0.0087	-0.55-0.02

Chl a		1.00-<0.0001	**0.97-<0.0001**	**0.96-<0.0001**	**1.00-<0.0001**
Chl b			1.00-<0.0001	**0.93-<0.0001**	**0.98-<0.0001**
Cars				1.00-<0.0001	**0.96-<0.0001**
Total chlorophyll					1.00-<0.0001
4 years-old bead	*Fluorescence chlorophyll*	*Chl a*	*Chl b*	*Cars*	*Total chlorophyll*
Fluorescence chlorophyll	1.00-<0.0001	0.31-0.24	0.33-0.20	0.35-0.19	0.32-0.23
Chl a		1.00-<0.0001	**0.96-<0.0001**	**0.97-<0.0001**	**1.00-<0.0001**
Chl b			1.00-<0.0001	**0.95-<0.0001**	**0.97-<0.0001**
Cars				1.00-<0.0001	**0.97-<0.0001**
Total chlorophyll					1.00-<0.0001
